# Ant Colony Optimization Algorithm for Interpretable Bayesian Classifiers Combination: Application to Medical Predictions

**DOI:** 10.1371/journal.pone.0086456

**Published:** 2014-02-03

**Authors:** Salah Bouktif, Eileen Marie Hanna, Nazar Zaki, Eman Abu Khousa

**Affiliations:** 1 Software Development, College of Information Technology, United Arab Emirates University (UAEU), Al-Ain, UAE; 2 Intelligent Systems, College of Information Technology, United Arab Emirates University (UAEU), Al-Ain, UAE; 3 Intelligent Systems, College of Information Technology, United Arab Emirates University (UAEU), Al-Ain, UAE; 4 Enterprise Systems, College of Information Technology, United Arab Emirates University (UAEU), Al-Ain, UAE; Rutgers University, United States of America

## Abstract

Prediction and classification techniques have been well studied by machine learning researchers and developed for several real-word problems. However, the level of acceptance and success of prediction models are still below expectation due to some difficulties such as the low performance of prediction models when they are applied in different environments. Such a problem has been addressed by many researchers, mainly from the machine learning community. A second problem, principally raised by model users in different communities, such as managers, economists, engineers, biologists, and medical practitioners, etc., is the prediction models’ interpretability. The latter is the ability of a model to explain its predictions and exhibit the causality relationships between the inputs and the outputs. In the case of classification, a successful way to alleviate the low performance is to use ensemble classiers. It is an intuitive strategy to activate collaboration between different classifiers towards a better performance than individual classier. Unfortunately, ensemble classifiers method do not take into account the interpretability of the final classification outcome. It even worsens the original interpretability of the individual classifiers. In this paper we propose a novel implementation of classifiers combination approach that does not only promote the overall performance but also preserves the interpretability of the resulting model. We propose a solution based on Ant Colony Optimization and tailored for the case of Bayesian classifiers. We validate our proposed solution with case studies from medical domain namely, heart disease and Cardiotography-based predictions, problems where interpretability is critical to make appropriate clinical decisions.

**Availability:**

The datasets, Prediction Models and software tool together with supplementary materials are available at http://faculty.uaeu.ac.ae/salahb/ACO4BC.htm.

## Introduction

Classification is a pattern recognition task that has applications in a broad range of fields. It requires the construction of a model that approximates the relationship between input features and output categories. The inputs describe several attributes of an entity that can be an object, a process or an event, and the outputs represent a set of classes to which the entity can belong. Typically, classification models are used to predict the class of new input data describing a previously-unseen entity. Although they are useful tools to support the decision-making process in their application fields, they still suffer from several limitations. One of the major problems is the low performance of a classifier when applied in new circumstances. The accuracy of a classifier could vary enormously from one dataset to another since a classifier that has produced good predictions for some datasets is not guaranteed to keep the same performance for other datasets [1]. This is due to the variation of data which typically follows the variation of the environment. This problem is worsened by the lack of representative data on the one hand and by the drawbacks inherited from the used modeling techniques on the other hand. Many methods have been dedicated to improve the performance of prediction classifiers when applied to new unseen data. Among these methods are the classifier ensembles by which a set of classifiers is combined to derive a final decision. Those methods are able to achieve a higher variance and a lower bias of the classification function realized by the collaboration of a set of involved classifiers [2].

Besides the performance problem, the utilization of classifiers in many fields suffers from the difficulty of interpreting the produced decisions. By interpretation, we mean the ability of a classifier (i.e., prediction model) to explain its predictions and exhibit the causality relationships between the input features and the output categories. This quality of classifiers is of a critical importance, especially when the user needs to focus his/her effort on improving some input features to prevent undesirable outputs. Therefore, with establishing a clear and explicit link between the predictor input features and the output decisions, the user can easily understand the effect of predictors variations and subsequently take the right actions on the input features. This understanding is important because it gives an insight into the work process in many domains. For example, in software engineering, the transparency of learned knowledge allows software engineer to know how faults originate during the development process and assists in taking the remedial actions [3]. In the context of software quality prediction, Andrew et al. [4] emphasize the fact that without clear semantics attached to a prediction model, the latter can not reach a satisfactory level of validity. With similar motivation, Fenton [1], [5] has qualified the models without easy interpretation as naïve and has proposed the use of Bayesian Networks (BN) as they are easily interpretable models.

In medical domain, the application areas of prediction models include diagnosing tumor malignancy, estimating the risk of cardiovascular disease, diabetes, pregnancy failure, tumor recurrence, estimating the therapeutic effect of different therapies, and detecting predictive factors for various conditions. All these applications are used in daily clinical practice to solve a broad range of clinical questions to guide clinicians when deciding upon the appropriate treatment and estimating patient-specific risks. Such clinical questions can not be answered without having a meaningful insight into the associations between explanatory variables and the dependent variables. Besides, with understandable models the resulting transparent diagnosis and risk estimate can be presented to the patient in a more comprehensible way than any advanced (i.e., complex) mathematical diagnostic models [6]. Moreover, in the modern schools of medicine, the comprehensibility of model enables a better doctor-patient communication, which is a very important goal in the age of informed patient decision making. In the field of drug discovery, not only the classification of the biological activity of a molecule is targeted but also the identification of the conformers responsible for the observed bioactivity for each molecule, is crucial [7]. Likewise, the ability to interpret prediction models is still one of the primary objectives in real-world business applications, where those models serve as tools to uncover relationships and identify the key variables influencing the classification outcome and the decisions.

In this paper, we propose a new method of classifiers combination based on Ant Colony optimization (ACO) and tailored for the case of Bayesian classifiers. The proposed method promotes performance and preserves the interpretability of the resulting prediction model. This method is validated with two different problems from the medical domain, namely, heart diseases and Cardiotography-based predictions. The main contributions and innovations of this paper are:

- A new implementation of classifiers combination approach that enhances the prediction performance.
- Customization of an emergent search technique, namely Ant Colony Optimization (ACO), on the problem of classifiers structure combination.
- Construction of composite classifier that preserves the ease of interpretability of individual Bayesian classifiers.
- Successful application of the interpretable classifiers combination on two different prediction problems from the medical domain.

### Related Work

In this section, we present the main ideas proposed in the literature to circumvent the problems of prediction models (e.g. classifiers), namely the low performance and the lack of interpretability issues. The efforts devoted to solve these problems fall under one of the following strategies. The first one aims at improving the predictive accuracy by reusing a set of single classifiers in order to derive a final decision from many individual predictions. This strategy is dominated by the methods known as Classifiers Ensembles. The second strategy aims at preserving an easy interpretation of the classifier decisions. This is achieved by choosing appropriate modeling techniques that derive “ white box” classifiers having the capacity to explain the causal relationship between inputs and outputs. As part of the first strategy, the Ensemble Classifiers Methods (ECM) have been widely applied to various real-word problems. They demonstrated that the combination of classifiers often outperforms the individual ones when it is applied on new data (e.g., [8]–[11]). In general with ECM, the individual classifiers are combined in different ways to derive a final output. These ways commonly include averaging, boosting, bagging and voting. Averaging consists in constructing a normalized weighted sum of *N* individual classifiers outputs (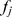).
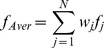
where 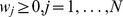 is the weight of 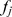. The weight 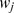 of an individual classifier can be interpreted as our confidence in the 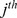 classifier. The simplest version of averaging is when the weighting is uniform (i.e., 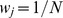), known as simple averaging [12]. The major intuitive benefit of averaging is achieved by reducing the estimate variance of the output error. Because of their simplicity, many improved versions of averaging have been proposed and used in different disciplines to provide a better prediction accuracy [11], [13]–[15]. Stacking mainly consists in combining multiple classifiers in two phases. In the first phase, 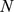 classifiers 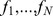 are built by using different learning algorithms 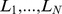 on a single dataset 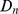. The training process of each individual classifier 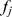 involves using a leave-one-out cross validation in which one data point 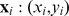 from 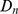 is left for testing. Leave-one-out cross validation method is deemed the most rigorous among others and hence it has been widely adopted by researchers [16]. In the second phase, the individual classifiers are applied on the set of left data points 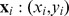 and a new dataset 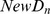 is built-up from 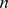 data points (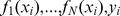). Each data point of 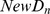 consists of 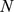 predictions of the individual classifiers in addition to the real class 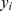 of a left data point. A final but important step is to learn a new classifier from the formed training dataset 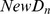. Issues of choosing the features and the learning algorithms have been discussed and solutions based on linear regression, multiple linear regression, decision tree, etc. have been proposed to learn the final classifier.

Boosting technique [17], iteratively produces a series of classifiers 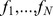, using a learning algorithm 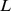 on a dynamically weighted dataset 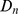. Each new classifier 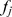 is built on a dataset 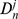, where its data points are weighted based on the performance of the precedent classifiers in the series 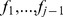. Obviously, the weights of previously misclassified data points are increased and the weights of correctly classified data points (i.e., by earlier classifiers) are decreased. Intuitively, the harder a data point is to learn, the higher is its new weight and vice-versa. In other words, the previously misclassified data points are given more chances to be correctly classified in the new classifier. AdaBoost is the most known algorithm that implements the boosting technique in the case of binary classification [17].

Another way to pool classifiers is Bagging. It starts by generating a random number 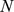 of subsets from the original training set. Then it utilizes them to learn individual classifiers 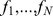. The training subsets, called bootstraps (i.e., sampled by replacement), are supposed to have enough differences in order to induce diversity among the individual classifiers. With Bagging, also called bootstrapped aggregating, the new data points are assigned the class that gets the maximum number of votes from the individual classifiers 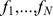. Voting can itself be considered as the simplest ECM technique [18], which assigns the class chosen by the majority of individual classifiers to a given example.

As a part of the second strategy that aims at promoting prediction interpretability, many researchers have devoted their works to show how critical it is to explain the prediction outputs. As mentioned above, this strategy mainly relies on preferring the utilization of particular modeling techniques such as decision trees, Bayesian Networks and Bayesian classifiers, rule set systems and fuzzy rules. Indeed, decision trees have been widely used as the most popular and interpretable modeling technique in economical, medical and engineering domains [4], [19], [20]. With the same motivation of supporting intuitive interpretation, Bayesian classifiers and Bayesian networks were used in several contexts including clinical diagnosis [21], text and mail classification [22], software engineering [23], etc. In particular, Fenton [1], [24] criticized existing techniques of software quality prediction because of the lack of interpretability. He described them as naive and proposed Bayesian models as highly interpretable thanks to the explicit causality links between features. Other modeling techniques were proposed to promote prediction interpretability in different domains. For example, in medicine the Interval Coded Scoring System [25] was used to identify patient-specific risks and Fuzzy rule-based models were used to diagnose the causes of coronary artery disease [20]. In the environmental management domain, rule-based models were created in order to define a better management of an ecosystem [26]. Adaptive fuzzy modeling was used in the process control engineering field to decide the level of molten steel in a strip-casting process [27].

In spite of the great efforts spent in the above strategies, the target of simultaneously promoting the performance and the interpretability of model prediction is rarely achieved. When the model performance is the goal, researchers, mainly from the machine learning community, tend to use all the possible mathematical justifications and techniques to increase the model performance. They take advantage of the diversity, availability and re-usability of different models to mathematically enrich a composite prediction. The tools range from simple weighted sum using constants to complex data-dependent weighted sum, and from simple learning from a simple dataset to iterative and incremental learning of models and weights from weighted datasets. The use of these techniques increases the complexity of the model and subsequently accentuates the “black box” property of the prediction process. Such a black box property of the ECM-based approach makes the interpretability hard and in many cases, worsens the interpretability of the original classifiers. In the second strategy, when the goal is to increase the interpretability, researchers, mainly working on application domains of prediction models, tend to trade the high performance of the models with a higher level of their interpretability. They tend to avoid the use of the ECM-based approaches, simply because the “white-box” property of the original classifiers is reduced in the sense that there is more than one classifier responsible for each decision.

Our proposal is a halfway technique. It is inspired by ECM but preserves the interpretability of the original classifiers. In this paper, we aim at circumventing two problems namely, the low performance, known in some application domains as generalization, and the interpretability preservation, also known as the “black-box” property of the prediction classifiers. This paper partly extends our previous work presented in [28] by giving more importance to the interpretability of classifiers. We propose a new classifiers combination scheme using the ACO algorithm; by increasing the interpretability of the resulting models; and by applying the developed approach in areas other than Software Engineering, namely in medical prediction problems.

### Problem Statement

As explained, a classifier relates its inputs representing the attributes that can be measured *a priori* and its outputs representing attributes that cannot be measured *a priori* but rather need to be predicted. For example, a prediction classifier for Heart Disease (HD) is built to predict the presence of HD in a patient by using a number of measurable symptom attributes such as blood pressure, chest pain type, etc. In the particular case where the prediction model is a classifier, the former is generally built/validated empirically using a data sample 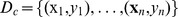 containing 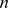 examples or data points, where 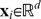, is an observation vector of 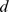 measurable attributes and 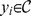 is a label to be predicted. The vector 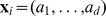 is the result of measuring 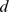 attributes. We let 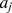 to be the generic value assumed by the 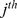 attribute.

The dataset 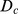 should be a representative sample of the data used for prediction. In the problem of HD, for example, the set 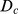 represents HD information of a patient population. This data characterizes a *particular context* of Heart Disease conditions that may bury not-yet-discovered HD risk attributes. In other words, patients from a particular HD context may share the same lifestyle, and the same nutritive traditions. With this same perception, if we want to take into account all the patient populations, we have to consider collecting data from many countries. For the sake of discussing some prediction solutions, let 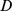 be the hypothetical set of all contexts overall the world.

To build a prediction model/classifier for particular circumstances using a context data 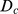, three alternatives can be considered: (1) applying a statistic-based or machine learning algorithm on 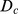, in this alternative only one context is considered which makes the resulting model unstable because the coverage of the set 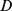 is low; (2) the second alternative consists in selecting the best available individual model using 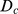, in this alternative, two contexts are used, however the coverage of the set 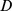 remains low; (3) the third alternative consists in reusing and eventually adapting as many existing models as possible using 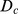 to guide a search process to collect the best chunk from each model. We believe that the third alternative is more valuable. Indeed, an ideal prediction model is a mixture of two types of knowledge: domain common knowledge and context specific knowledge. By reusing existing models, we reuse the common domain knowledge represented by versatile contexts. Intuitively, when more contexts are covered, the resulting prediction model is more generalizable. On the other hand, by guiding the adaptation via the context specific data, we take into account the specific knowledge represented by 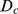. Subsequently, by adapting and reusing multiple chucks of expertise, we target the goal of building an expert that outperforms all the existing models (i.e., the models that are already built by third party or by using an available dataset collected for the sake of controlled experiment). The *best expert*, i.e. the existing model achieving the highest accuracy on the dataset 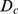 in turn, will play the role of a benchmark for evaluating our proposed solution.

In the present work, we consider the problem of reusing 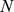 predefined prediction models 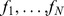 called *experts*. In particular, we propose a new particular solution for combining Bayesian Classifiers (BC). The challenging question is how to produce a new optimal BC that inherits the “white-box” property, i.e. ease of interpretation of BCs, while improving the accuracy, i.e. the ability of generalization on the available context represented by 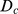. These BCs will be considered as experts.

### Method

To avoid the drawbacks of traditional combination methods (see Section 2), we propose an approach that reuses the existing classifiers to derive new classifiers having higher predictive accuracies, without worsening the interpretability of the original experts. Considering this objective our approach consists of three principles:

*Principle 1* decomposes each expert into chunks of expertise. Each chunk represents the behavior of the expert on a “partition” of the entire input space (i.e., the whole hypothetical dataset 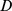). In general, a chunk of expertise can be defined as a component of the expert knowledge, which can be represented using certain techniques such as linear regressions, decision trees, Bayesian classifiers, etc. The “partitioning” of the input space depends on the structure of the expert representation. For example, the decomposition of a decision tree leads to expertise chunks in form of rules, thus a “partition” is a decision region in the input space. However, for a Bayesian classifier, the decomposition yields expertise chunks in the following way: each attribute is subdivided into intervals, to each interval (i.e., range of attribute values) is attached a set of conditional probabilities (See more details in Section 4.2.1).The rational behind the first principle is to give more flexibility to the process of combination, specially when selecting the appropriate chunk of expertise. Therefore, an expert might have some accurate chunks of expertise although its global performance is low and vice-versa. Moreover, the derived expert which is a combination of chunks of expertise will be interpretable since we know the chunks that are responsible for the final decision.

*Principle 2* reuses the chunks of models coming from different experts in a way to progressively build more accurate combinations of expertise using 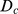 to guide the search.

*Principle 3* modifies some chunks of expertise in order to obtain new combinations of expertise that are more adapted to the particular context 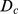.

This three-principle process of building an optimal expert can be thought of as a searching problem where the goal is to determine the best set of expertise suitable for the context 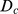. Several available experts will be decomposed into a set of expertise chunks. The combination of these expertise will generate a combinatorial explosion which makes the problem an NP-complete one. Such a problem can commonly be solved by using a search based technique in a large search space. In the current solution of combining Bayesian classifier experts, we propose a customization of the ACO as a promoting technique to implement our approach.

### 4.1 Naïve Bayesian Classifier

A Bayesian classifier is a simple classification method, that classifies a 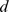-dimensional observation 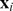 by determining its most probable class 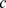 computed as:
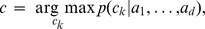
where 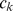 ranges of the set of possible classes 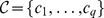 and the observation 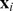 is written as generic attribute vector. By using *the rule of Bayes*, the probability 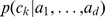 called probability *a posteriori*, is rewritten as:
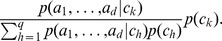



The expert structure is drastically simplified under the assumption that, given a class 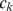, all the attributes are conditionally independent. Accordingly, the following common form of *a posteriori* probability is obtained:
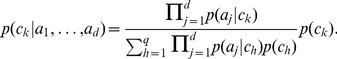
(1)


When the independence assumption is made, the classifier is called Naive Bayes. 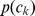 called marginal probability [1], is the probability that a member of a class 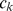 will be observed. 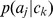 called prior conditional probability, is the probability that the 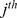 attribute assumes a particular value 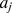 given the class 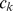.

A naive BC treats discrete and continuous attributes in different ways [29]. For each discrete attribute, 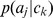 is a single real value that represents the probability that the 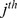 attribute will assume a particular value 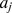 when the class is 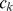. Continuous attributes are modeled by some continuous distribution over the range of that attribute’s value. A common assumption is to consider that within each class, the values of continuous attributes are distributed as a normal (i.e., Gaussian) distribution. This distribution can be represented in terms of its mean and its standard deviation. Then we interpret an attribute value 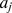 as laying within some interval. The attribute domain is divided into 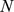 intervals 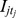 and 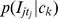 will be the prior conditional probability of a value of the 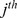 attribute to be in the interval 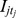 when the class is 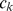; 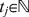 is the rank of the interval in the attribute domain. To classify a new observation 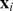 (i.e., 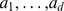), a naïve BC with continuous attributes applies the Bayes theorem to determine the *a posteriori* probability as:
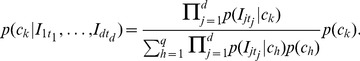
(2)with 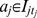.

### 4.2 ACO Based Approach

Ant Colony Optimization algorithm was inspired by the biological behavior of ants when looking for food. This behavior was closely observed and investigated in [30]. The process by which ants search for food and carry it back to their nest is very efficient. Throughout its trip, an ant deposits a chemical substance called pheromone which is usually used as a mean of indirect communication between species members [31]. The amount of pheromone deposited by an ant reflects the quality of the food and the traversed path. Observations show that in the beginning of the food search, the ants randomly choose their paths. Nevertheless, after some time and based on their communications through pheromone trails, they tend to follow the same optimal path. A graph in which the set of possible solution components can be modeled as vertices or edges is used to represent an optimization problem. Based on this representation, an artificial ant builds a solution by moving along the graph and selecting solution components. The deposited amount of pheromone mirrors the quality of built solutions.

Like other metaheuristic techniques, ACO has to be customized to the particular problem we are solving. We recall that we want to exploit ants’ foraging behavior to derive an optimal set of expertise that performs well on a given context represented by the dataset 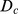. Deriving an optimal expert will not only include the selection of existing expertise chunk from the original Bayesian classifiers, but also the creation of new chunks of expertise mutated from existing ones. The work of the artificial ants will then consist of reusing and creating combinations of expertise.

The customization of ACO to the Bayesian classifiers combination problem needs the definition of the following elements: a solution representation, a graph on which the artificial ants will construct the solutions, a measure of the solutions accuracy, a suitable strategy for ant communication via pheromone update and finally a moving rule based on which ant decides to move from one node to the next in the graph [32].

#### 4.2.1 Solution Representation

The partitioning of a BC into chucks of expertise is central to our approach. This operation facilitates the exploration of the search space defined by all the combinations of original and modified chunks of expertise. Consequently, it makes the steps of reusing and adapting the existing BCs easier and efficacious.

According to the description of Naïve BCs given in Section 4.1, two kinds of parameters of a BC can represent a chunk of expertise. The first is the marginal probabilities of different classes 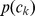, where 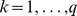. The second is the prior conditional probabilities of the attributes 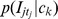. Since the prior conditional probabilities are more relevant to express a different structure for a BC, they are chosen to characterize a chunk of expertise.

To each attribute 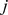, 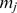 chunks of expertise are associated. A chunk of expertise can be represented by a 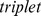 made up of an interval and two conditional probabilities. To illustrate the interpretation of a chunk of expertise, let us consider the prediction of Heart Disease (HD) prediction problem. The used BCs are binary and predict either the presence or the absence of heart disease in a patient’s body. The set of class labels is 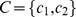, with 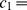 *PresenceHD* and 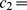 *AbsenceHD*. In this example a chunk of expertise 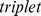, denoted by 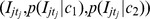, can be interpreted as follows: the prior conditional probability of a value of the 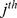 attribute to be in the interval 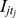 when the class is 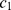, is equal to 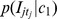 and 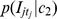 when the class is 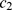. The index 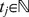 is the rank of the interval in the attribute domain containing 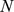 intervals. Continuing with the same prediction problem, an HD symptom attribute 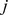 (e.g., the 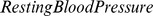) in a Bayesian classifier will be represented by the following structure:
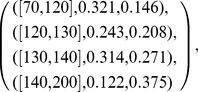



where each line is a triplet 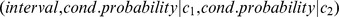 that encodes a HD chunk of expertise. For example, the HD expertise defined by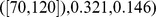 means that the conditional probability, of a value of *RestingBloodPressure* to be in the interval 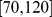 when the class is 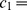 *AbsenceHD*, is equal to 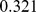 and 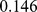 when the class is 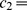 *PresenceHD*. Note that this symptom attribute *RestingBloodPressure* is divided into 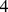 intervals.

#### 4.2.2 Ant solution construction mechanism

Using ACO, two strategies of modeling BCs combination are possible. The first is inspired by the modular structure of BC, in which we propose to apply ACO on each single attribute. In other words, our artificial ants will iteratively construct a new composition for each attribute until obtaining a near optimal set of expertise. The work of the ants on each attribute, will consist in deriving, a new slicing of the attribute domain and a new distribution of the conditional probabilities. This process is separately repeated for all the attributes in a parallel or a sequential manner. Then, a final classifier is built-up by grouping the obtained near-optimal compositions of all the attributes. The second strategy aims at iteratively constructing new BC solutions until obtaining a near optimal one. Within this strategy, at each iteration, the work of the artificial ants consists in simultaneously constructing chunks of expertise for all the attributes, in order to derive new BCs. Knowing that both strategies have advantages and disadvantages, in this paper we will explore the first strategy and will empirically study and compare the two strategies in our future work. Accordingly, we focus on applying the ACO at the BC attribute level.

Combining attributes can be modeled as a search problem of an optimal path in a directed graph 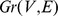 where 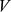 is a set of vertices and 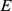 a set of edges. A main task of the ACO customization consists in constructing the graph on-which the ants will build the solution.

#### 4.2.3 Attribute graph construction

We define an instance of the attribute 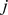 as its composition in terms of intervals in a particular BC. This composition can be represented by a sorted vector of boundaries of those intervals. Since we have 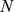 BC to be combined, each attribute 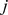 has, accordingly, 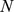 instances. Hence, in order to construct the attribute graph 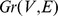 of an attribute 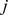, we first consider all the instances of the attribute 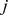. This step consists in forming a composite sorted vector that holds all the boundaries got from all the instances of the attribute 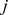. This composite vector is used to create the new composite instance of the attribute 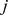, in which, each interval is bounded by two consecutive values from the composite vector. Therefore, each vertex 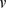 in 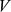, the set of vertices, represents a boundary from the composite vector. The order of nodes in the attribute graph is following the order of boundaries values in the composite vector. In the case of combining 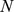 binary BCs, there are 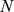 edges 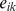, 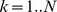, between two *consecutive* nodes 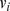 and 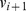. Each edge, 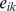 in 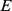, represents a couple of conditional probabilities (i.e., 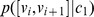, 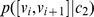) associated with the attribute interval 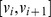. These probabilities are computed based on the conditional probabilities distribution of the original instance of attribute coming from the 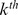 BC. For example, the conditional probabilities labeling the edge 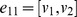 are computed in the following way:
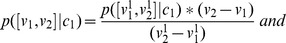


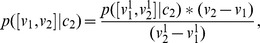
where, 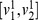 is the an interval from the original composition of the attribute 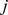 in the BC number 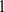.

**Figure 1:**
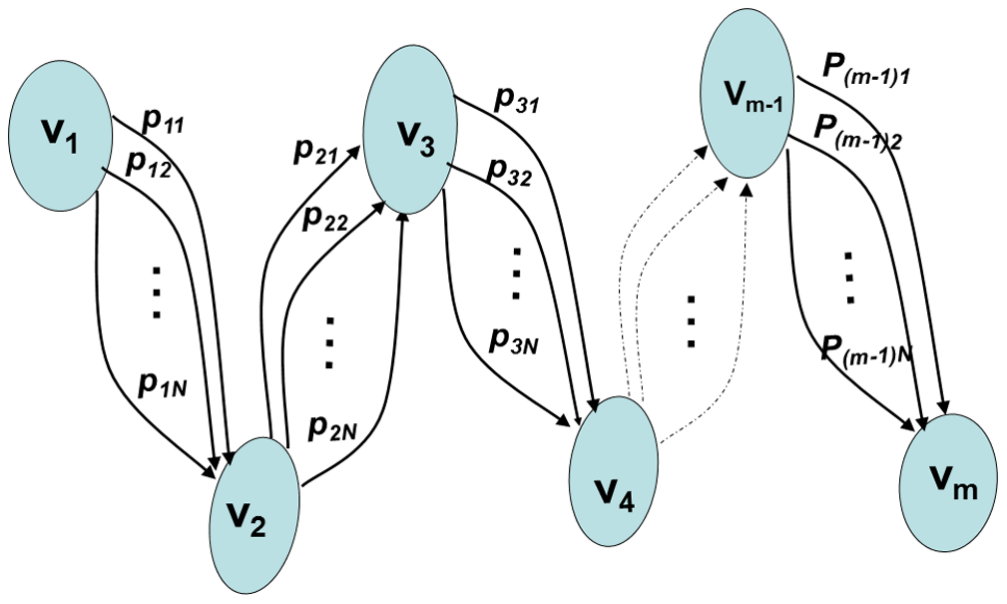
Graph for the Solution Construction Mechanism.

Figure 1 shows the graph constructed for an attribute 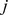. It depicts the new slicing (into intervals) of the attribute domain that takes into account the original compositions of the attribute 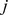 through 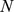 individual BCs. For any given vertex 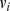, outgoing edges (incoming to 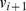) represent (i.e., are labeled by) all possible couples of conditional probabilities associated with the interval 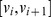 originating from 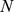 individual BCs involved in the combination process. For the sake of simplicity of the graph in Figure 1, the label 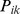 of an edge 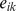 represents the couple of probabilities 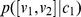 and 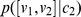 computed based on the original conditional probabilities of the interval 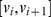 in the BC number 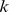.

#### 4.2.4 Solutions construction

As described above, the solutions construction mechanism assumes that the used graph (see Figure 1) is *static*, built on quantized pairs of conditional probabilities domain; all possible values of conditional probabilities are pre-determined, listed, and used to build the static graph. Thus, a candidate attribute solution is an instance constructed by traversing the attribute graph while following the nodes order from the first node (lower boundary of the attribute domain ) to the last node (upper boundary of the attribute domain). In each transition to next node one edge is selected to form at the end a combination of edges.

#### 4.2.5 Solution quality measure

We recall that we need to evaluate an attribute composition constructed by the ants. Every move of an ant has to be taken into account since it has an impact on the composition of the attribute being constructed. A new attribute composition has to integrate a BC in order to be evaluated. Thus, the accuracy of the subsequent new BC will indicate the quality of the attribute composition. During the execution of the ACO algorithm at the attribute level, we use a BC for which we fix all the attribute compositions but the one being evaluated. The mission of the ACO algorithm is to maximize the predictive accuracy of the BC by integrating the processed attribute. Our approach is a learning process where the dataset 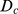 representing the particular context of prediction is used to guide the ants in their trails to construct solutions. Therefore, the set 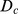 is used as an evaluation data set for computing the predictive accuracy of the classifier proposed by the ACO process at the attribute level.

This predictive accuracy of BC can be measured in different ways as discussed in [33]–[35]. An intuitive measure of it is the *correctness function* is given by:
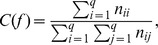
where 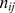 is the number of cases in the evaluation dataset with real label 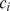 classified as 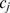 (Table 1). Note that for a BC, the class label 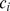 of a given case is the label that has the highest posterior probability (see equation 2).

**Table 1:** The confusion matrix of a decision function 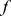. 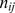 is the number of cases in the evaluation dataset with real label 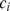 classified as 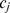.

		Predicted label			
		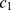	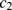	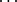	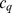
	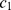	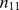	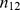	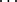	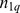
real	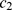	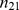	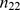	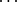	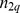
label	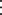	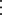	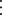	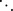	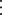
	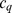	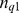	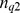	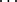	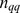

In several prediction problems, the data is often *unbalanced*; for example, patients tend to be healthy and not suffering from heart diseases. A much higher probability is assigned to the majority class labels. On an unbalanced dataset, low training error can be achieved by the constant classifier function 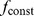 that assigns the majority label to every input vector. To give more weight to data points with minority class labels, we decided to use Youden’s *J-index* [36] defined as
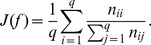



Intuitively, 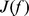 is the average correctness per label. If we have the same number of points for each label, then 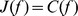. However, if the dataset is unbalanced, 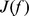 gives more relative weight to data points with rare labels. In statistical terms, 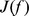 measures the correctness assuming that the *a priori* probability of each label is the same. Both a constant classifier 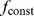 and a guessing classifier 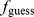 (that assigns random, uniformly distributed labels to input vectors) would have a J-index close to 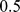, while a perfect classifier would have 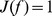. For an unbalanced training set, 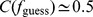 but 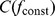 can be close to 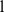.

#### 4.2.6 Ant walk, attractiveness and visibility

Using the graph attribute 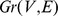, at each iteration of the ACO algorithm, all ants start their trails from the vertex representing the lower boundary, 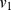, of the attribute, complete one tour visiting all vertices and finish at the vertex representing the upper boundary of the attribute domain. When an ant on a vertex 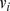 moves to the next vertex 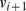, it chooses an edge 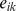 representing the 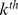 couple of conditional probabilities associated to the interval 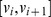 and originally yielded from the 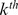 BC. In other words, the ant’s task after each move, is to assign a pair of conditional probabilities to an attribute interval 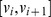.

At the beginning, the ants start by moving randomly from one vertex to the following one. In the following iterations, these moves are guided by a certain transition strategy. Actually, the choice of an edge to be traversed depends on the amount of pheromone accumulated on that edge. The higher the amount of pheromone, the higher will be the probability of choosing that edge. This probability is defined by the following equation:
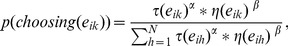
(3)where 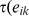 and 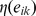 are respectively the attractiveness and the visibility of the edge 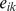 to be chosen. The attractiveness function is based on the success of the previous solutions. It is modeling the amount of pheromone accumulated on the trail of an ant is defined in Equation 4. However, the visibility function is defined as the sum of conditional probabilities associated to the edge 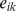. This definition is inspired by analogy to path minimization problem where the visibility, is reciprocal of the distance between the two nodes of the edge. In the proposed definition of edge visibility, the higher the sum, the more visible is the edge. The two parameters 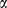 and 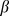 are used to balance the impact of attractiveness (i.e., pheromone) versus visibility. These are two parameters of the ACO Algorithm and have to be set empirically after several runs. After calculating the probability of choosing for every edge 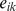, 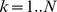, a Casino wheel selection method is applied to determine the chosen edge.

#### 4.2.7 Pheromone update strategy

When an ant traverses an edge, it deposits a pheromone amount on it. The accumulated amounts of pheromone form the attractiveness of an edge. This can also be interpreted as a *long-term memory* of the ant colony. In our proposed ACO algorithm, this long-term memory is updated each time an ant finishes one tour. The strategy of updating the pheromone amount deposited, in the iteration 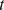 on an edge 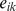, 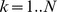, is governed by the following equation:
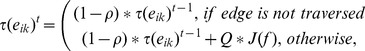
(4)where 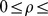 is a parameter of the ACO algorithm representing the evaporation rate of the pheromone substance. A small value of this parameter means that the evaporation is low and vice-versa. The 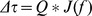 is the newly deposited pheromone that contains the base attractiveness constant 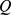 and a quality measure 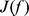 to be maximized. The accuracy 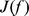 is measuring the quality of a HD Bayesian classifier 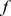 containing the attribute being treated (see Section 4.2.5). The process is iterated and at each tour increasingly accurate attribute compositions are constructed until a stopping criterion is met.

### Experimental Works

The applicability of our approach is not restricted to one particular domain. In this paper, we evaluate the proposed ACO based approach of combining Bayesian classifiers by conducting controlled experiments, on two problems from the medical domain where data is available. The chosen two problems are namely, the Heart Disease prediction (HD problem, for short) and Cardiotocography-based fetal pathologies prediction (CTG problem). In the HD problem a prediction model tries to predict the presence or the absence of heart disease in a patient and in the CTG problem, a prediction model tries to predict potential fetal pathologies. For both problems, interpretability is increasingly gaining high interest. In fact, heart disease is considered by the World Health Organization (WHO) as the leading cause of death in many world-wide populations [37]. In Japan for instance, the number of strokes has fallen by more than 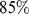 when the government has discovered that the trigger for heard disease is blood pressure [38]. How has the Japanese government been successful in achieving this impressive reduction of the number of strokes in its population? The answer to this question highly valued the preventive actions of health screening and education. Several studies have been done in University of Osaka to discover how risk factors contribute to strokes [38]. The interpretation of the relationship between a stroke and its risk factors, guided the government to focus their efforts on establishing community-based programmes including regular health check-ups to control key risk factors and health promotion campaigns on healthy lifestyle. With respect to the CTG problem, the cardiotocography is used for electronic fetal monitoring in order to record during pregnancy, the fetal heart beat, uterine contractions, etc. The continuous monitoring by using CTG requires interpretations of several features as described in Table 3 in order to predict potential fetal pathologies. The ultimate goal of the proposed approach is to build a high performance and interpretable prediction model. To achieve this goal, two datasets are used: (1) A set of existing models called experts and (2) a representative dataset that will be used to guide the combination process of the experts, called context data.

### 5.1 Data Description

#### 5.1.1 Data for HD problem

For the sake of results validity, three separate datasets representing three different populations of HD patients and collected in three different locations, are used in our experiments. These datasets were freely available from UCI machine learning repository [39]. Table 2 summarizes the properties of datasets used in the three experiments on HD problem.

**Table 2:** Dataset description.

dataset Name	Location	Size	Reference
*Cleveland*	Cleveland Clinic		[45]
	Foundation, Ohio		
*Hungarian*	Hungarian Institute		[46]
	of Cardiology, Budapest		
	V.A. Medical Center,		[41]
*Long Beach*	Long Beach, California		

Each dataset uses 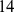 symptom attributes of HD selected out of an original set of 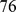 attributes. The selection of the 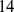 attributes was a consensus of machine learning researchers in several previous published experiments such as in [40] and [41]. Accordingly, every patient from the studied three populations is described by a vector of 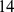 values, 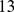 of them are mapping symptom attributes and one is a binary variable equal to 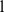 when the patient has HD and 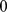 otherwise. The 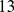 attributes are then used as inputs of the simulated HD experts. A description of these symptoms attributes is given by Table 3.

**Table 3:** The 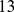 symptom attributes used to predict HD in the experiment.

Name	Description
AGE	age of a patient
SEX	sex of patient (1 = male; 0 = female)
CPT	Chest Pain Type
	– Value 1: typical angina
	– Value 2: atypical angina
	– Value 3: non-angina pain
	– Value 4: asymptomatic
TRESTBPS	: resting blood pressure (in mm Hg on admission to the hospital)
CHOL	Serum Cholesterol in mg/dl
FBS	(Fasting Blood Sugar 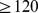 mg/dl) (1 = true; 0 = false)
RESTECG	Resting Electrocardiographic results
	– Value 0: normal
	– Value 1: having ST-T wave abnormality (T wave inve-
	rsions and/or ST elevation or depression of 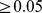 mV)
	– Value 2: showing probable or definite left ventricular
	hypertrophy by Estes’ criteria
THALACH	maximum heart rate achieved
EXANG	exercise induced angina (1 = yes; 0 = no)
OLDPEAK	ST depression induced by exercise relative to rest
SLOPE	the slope of the peak exercise ST segment
	– Value 1: up-sloping
	– Value 2: flat
	– Value 3: down-sloping
CA	number of major vessels (0–3) colored by fluoroscope
THAL	3 = normal; 6 = fixed defect; 7 = reversible defect

#### 5.1.2 Data for CTG problem

The dataset used for the CTG problem is published in the UCI repository and collected by the faculty of Medicine at the University of Porto, Portugal [42]. It contains 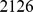 records of fetal cardiotocographies represented by 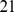 diagnostic attribute related to fetal heart rate and uterine activity. These attributes are inputs of a binary classifier that distinguishes normal fetal cardiotograms from pathological ones. A short description of the CTG attributes is shown in Table 4.

**Table 4:** The 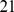 CTG attributes used to predict potential fetal pathologies.

Name	Description
FHRBL	Fetal Heart Rate (FHR) Baseline (beats per minute)
AC	# of accelerations
FM	# of fetal movements per second
UC	# of uterine contractions per second
DL	# of light decelerations per second
DS	# of severe decelerations per second
DP	# of prolonged decelerations per second
ASTV	percentage of time with abnormal short
	term variability
MSTV	mean value of short term variability
ALTV	percentage of time with abnormal long
	term variability
MLTV	mean value of long term variability
Width	width of FHR histogram
Min	minimum of FHR histogram
Max	maximum of the histogram
Nmax	# of histogram peaks
Nzeros	# of histogram zeros
Mode	histogram mode
Mean	histogram mean
Median	histogram median
Variance	histogram variance
Tendency	histogram tendency: −1 = left assymetric;
	0 = symmetric; 1 = right assymetric

### 5.2 Individual Experts “Construction” and Context Data

Although, the proposed approach assumes the availability of already built experts, we chose to perform a controlled experiment in which the individual experts were built “in-house”. Two thirds of each dataset was used as training data to build a number of experts, which simulate the existing prediction models. Accordingly, in the case of HD problem, we obtained three training datasets, respectively referred to as 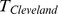, 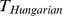 and 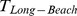. The remaining one-third of each dataset is used to form the context data representing the HD diagnosis of a particular patients population. The context data of a population is used to guide the combination process in order to derive a prediction model appropriate for such population conditions. We respectively, form three context datasets referred to as 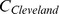, 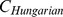 and 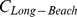. Similarly, in the case of CTG problem, we created a training dataset referred to as 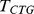 and a context dataset denoted 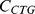.

From each training dataset and by using random combinations of attributes, we formed 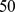 subsets of training data. By using a different combination of attributes in each subset of data, we imitated different opinions of experts of the targeted prediction problem. In addition, by randomly splitting each of the obtained datasets into two subsets, we created in total 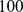 final training sets. Then, a classifier is trained on each training set by using the RoC machine learning tool (the Robust Bayesian Classifier, Version 1.0 of the Bayesian Knowledge Discovery project) [43]. Among the 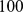 learned BCs, we retained the top ones having lower training errors (i.e., these are 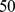 in the HD case and 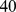 in the CTG case). The numbers 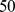 and 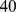 are the sizes of the smallest set of classifiers achieving a training error 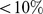 in the case of HD and in the case of CTG, respectively.

This procedure of building individual BCs is repeated for the three training datasets, 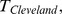 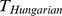 and 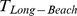, in the case of HD prediction problem and is also repeated for the training dataset 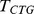 in the case of CTG-based prediction. Accordingly, 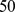 HD BCs are derived from the data of each HD population (*Cleveland, Hungarian* and *Long-Beach*), and 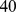 CTG BCs are built from the CTG data.

### 5.3 Experimental Design

To evaluate the performance of the resulting models of our approach, on the two studied problems, four independent experiments were conducted in order to build BCs for HD prediction and for CTG prediction. Three of the experiments are carried on the three different HD contexts, namely, 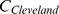, 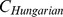, 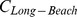. In each experiment, a composite HD BC was derived by combining individual BCs learned in two of the three contexts while being guided by the third one. In the fourth experiment conducted for the CTG problem, a composite CTG BC was built by combining individual BCs trained on 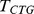 while being guided by 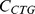. Table 5 specifies the two inputs of our approach for the four experiments.

**Table 5:** Experiments description.

Experiment#	Prediction	Individual BCs	Population
	Problem	learned on	(Context dataset)
1	HD	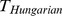 &	*Cleveland*
2	HD	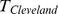 &	*Hungarian*
		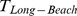	( 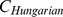 )
3	HD	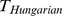 &	*Long-Beach*
		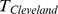	( 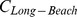 )
4	CTG	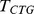	*Porto*
			( 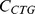 )

In each experiment, the accuracy of the resulting composite BC, named 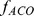, is compared to those of BCs built by other benchmark methods of improving model performance. Four of these methods were investigated: (1) selection of the best existing model, (2) combination of all training data (3) boosting method and (4) bagging method. The first two methods are intuitive and have the advantage of not worsening the model interpretability. The last two methods belong to the ensemble classifiers methods, known to be successful in achieving high model accuracy. The classifiers derived by these methods are, respectively, named 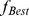, 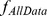, 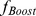 and 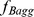. They are constructed within each of the four experiments in the following way:

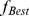
 : the best existing BC is determined after measuring the accuracy of the 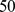 HD (*resp.* 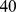 CTG) individual BCs, used as input models to our approach, on the context data of the experiment. Then 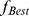 is the individual BC among the existing ones that has the highest accuracy on the considered context data.

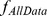
 : the individual BC derived from the data that has been used to build all the 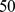 HD (*resp.* 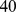 CTG) individual BCs. To construct this BC, the datasets that have been used to train the individual BCs (i.e., input models) are combined into one global dataset called 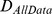. Then 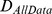 is used as a training set to build a new BC referred to as 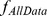. In HD prediction problem, the dataset 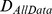 consists of the union of 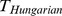 and 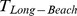 in experiment#1, the union of 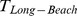 and 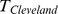 in experiment #2, and of the union of 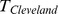 and 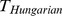 in experiment #3. However it is equal to 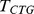 in the case of CTG prediction problem evaluated by experiment #4.

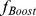
 : the classifier derived from combining the 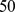 HD (*resp.* 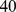 CTG) individual BCs using the well known Adaboost algorithm (more details on Adaboost are in Section 2).

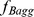
 : the classifier derived from combining the 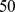 HD (*resp.* 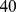 CTG) individual BCs using the bagging algorithm.

### 5.4 Hypotheses

To perform the above comparisons and to determine the right conclusions, we proposed a set of hypotheses to be tested for two different prediction problems (i.e., HD and CTG). In the four performed experiments, we assume that we are proposing an approach which, on the one hand, performs better than 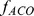 and 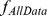, and on the other hand, is as good as ensemble classifiers based methods, such as Bagging and boosting. According to these assumptions, the following hypotheses were formulated and tested with four different contexts, namely, 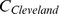, 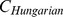, 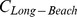 and 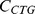 (See Table 5).

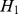
: The composite BC 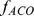, derived by ACO-based approach has a higher predictive accuracy than the best individual experts 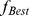.

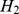
: The composite BC 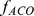, derived by ACO-based approach has a higher predictive accuracy than the expert, 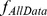, trained on all the data used to build the simulated individual experts.

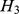
: The accuracy of the composite BC 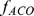, derived by ACO-based approach is at least as high as the accuracy of the classifier obtained by the Boosting ECM 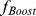.

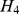
: The accuracy of the composite BC 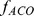, derived by ACO-based approach is at least as high as the accuracy of the classifier obtained by the Bagging ECM 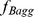.

### 5.5 Ant Colony Optimization Setting

In each experiment, the parameters setting of the ACO algorithm is determined based on several runs. The goal of the setting phase is to assign parameter values that allow high accuracy of the derived model without falling in the overfitting problem. Therefore, the termination criterion 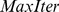, the number of artificial ants 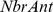, the pheromone variation 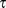, the pheromone evaporation rate 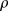, the impacts of pheromone 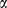, and the pheromone visibility 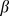 are set according to Table 6.

**Table 6:** ACO parameters setting.

Experiment#	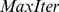	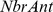	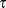	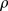	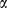	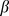
1	150	100	1.0	0.02	2.0	1.0
2	120	70	1.0	0.04	3.0	2.0
3	150	100	1.0	0.02	2.0	1.0
4	100	50	2.0	0.03	2.0	2.0

## Results

To verify the hypotheses for the four contexts, the accuracies of the obtained classifiers were evaluated using J-index of Youden (See Section 4.2.5) and estimated using 10-fold cross-validation. Accordingly in each of the experiments, the evolution of the ACO algorithm to derive a new BC is guided by the union of 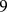 folds from the context data 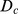. In other terms, a new BC 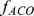 is then trained on the union of 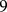 folds, and tested on the remaining fold. Similarly, the two classifiers 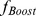 and 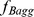, respectively derived by the boosting and the bagging algorithms are trained on the union of the same 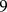 folds, and tested on the remaining fold. With respect to the first two benchmark approaches, the derived BCs 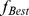 and 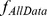 are simply evaluated on both the union 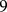 folds, and tested on the remaining fold. The whole process, i.e., for ACO and the alternative approaches, is repeated 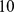 times for all 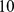 possible combinations. For each approach, the accuracy mean and standard deviation are calculated for J-index on both the training and the test samples. Results are obtained for the three HD contexts 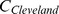, 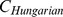 and 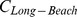 as well as for the CTG context 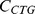. These are respectively, reported in Tables 6, 7, 8 and 10.

**Table 7:** Experimental results for HD prediction problem. Accuracy percentage values of ACO and Benchmark approaches in the context of *Cleveland* population, (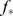 is the classifier compared to 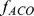).

	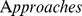				
	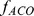	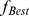	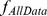	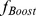	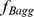
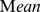	73.33	54.45	61.08	51.61	66.19
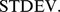	11.95	13.78	12.78	11.50	13.61
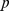 -value	–	0.003	0.040	0.001	0.23
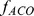 vs. 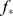		(Two-tail)			

**Table 8:** Experimental results for HD prediction problem. Accuracy percentage values of ACO and Benchmark approaches in the context of *Hungarian* population, (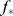 is the classifier compared to 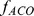).

	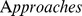				
	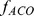	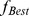	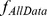	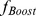	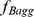
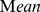	73.27	57.53	59.86	69.14	69.40
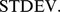	4.60	3.74	4.65	4.80	100
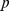 -value	–	0.007	0.039	0.514	0.476
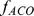 vs. 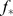		(Two-tail)			

**Table 9:** Experimental results for HD prediction problem. Accuracy percentage values of ACO and benchmark approaches in the context of *Long-Beach* population, (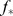 is the classifier compared to 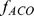).

	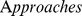				
	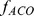	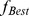	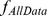	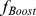	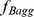
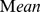	69.63	44.70	56.12	67.13	55.36
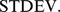	15.77	9.01	12.13	16.82	13.71
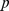 -value	–	0.0023	0.04	0.04	0.39
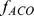 vs. 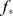		(Two-tail)			

**Table 10:** Experimental results for CTG prediction problem. Accuracy percentage values of ACO and benchmark approaches in the context of *CTG*, (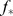 is the classifier compared to 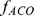).

	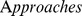				
	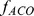	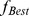	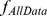	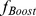	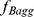
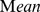	74.60	64.05	59.16	55.00	60.32
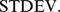	11.61	15.16	25.99	21.35	16.13
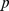 -value	–	0.049	0.056	0.011	0.018
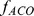 vs. 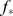		(Two-tail)			

### 6.1 Comparison with Best Expert

The obtained results for both HD and CTG predictions, show a considerable improvement in the accuracy of the generated BC when compared to the best expert 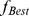. Indeed, in the three HD contexts as well as in the CTG context, the resulting BC, 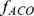 has gained between 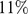 and 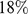 in predictive accuracy on the training dataset, and between 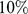 and 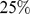 on the testing data. A statistical analysis of the results using 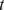-test shows that the null hypothesis 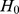, assuming that 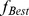 accuracy is not higher than the accuracy of 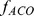, is rejected with a very strong evidence, greater than 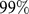 in all the three HD contexts and greater than 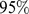 in the CTG context.

### 6.2 Comparison with Data combination

A similar comparison between the resulting BC 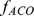 and the BC trained on all the available data denoted 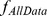 shows over all the HD and CTG contexts an accuracy increase achieved by 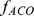 that ranges between 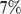 and 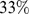 on training data, and between 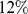 and 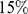 on testing data. A statistical testing using the 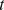-test shows a signicant difference between 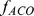 and 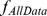. The null hypothesis 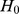, assuming that 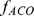 accuracy is not higher than that of 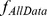, is rejected by 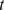-test with very high confidence greater than 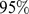 in the HD contexts as well as in the CTG context. (i.e., One-tailed 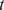-test 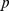-value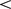 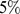 in all the contexts).

### 6.3 Comparison with Boosting

In comparison with the ECM based methods, it is noticed in the three contexts that our ACO approach preforms better than Boosting and Bagging. Indeed, in the case of HD prediction, 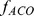 has achieved higher accuracy than 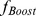 with gains ranging from 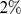 in the *Long-Beach*’s context to 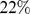 in the *Cleveland*’s one. The statistical analysis of the comparison results in the *Cleveland*’s context show that the null hypothesis 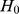, stating that 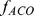 accuracy is lower than the 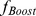 accuracy, is rejected at significance level of 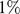 (i.e., 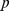-value = 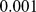). In both contexts *Hungarian* and *Long-Beach*, results show only a slight outperformance of 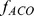 over 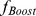 which explains why the statistical analysis fails to reject the same null hypothesis. However, our assumption, stating that our ACO-based approach is as at least as good as the Boosting methods, has held up. In the case of CTG prediction, 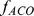 has outperformed 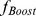 with an accuracy gains of 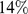 and 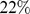 on the testing and training data, respectively. The statistical analysis of the comparison results in the *CTG*’s context show that the null hypothesis 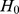, stating that 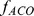 accuracy is lower than the 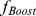 accuracy, is rejected at confidence level of 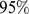 (i.e., 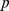-value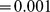).

### 6.4 Comparison with Bagging

More consistent achievements are noticed in the comparisons with Bagging approach (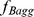) in all the experiments. In these comparisons, 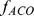 accuracy in *Hungarian*, *Cleveland*, *Long-Beach* and *CTG contexts, has respectively gained* 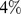 *,* 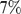 *,* 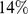 *and* 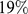 *on the testing data.* These results hold up our assumption that 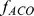 accuracy is at least as high as the accuracy of (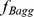). Moreover in the *Long-Beach* context, from HD problem as well as in the *CTG context from CTG problem*, the null hypothesis 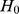, stating that 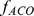 accuracy is lower than the 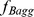 accuracy, is rejected using the 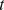-test at a significance level of 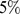 (i.e., 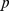-values 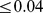).

## Discussion

The results obtained with the above comparisons support our claims about the proposed ACO-based approach. Indeed, it is evaluated against two different prediction problems represented by four different contexts (tree for HD prediction and one for CTG based prediction). Four benchmark approaches are compared to the proposed one and the summary of the results shows (1) a significant outperformance over both best expert and data combination approaches and (2) a comparable performance to ensemble classifiers methods (Bagging and Boosting). Nonetheless, some threats to validity has to be considered which may provide better interpretation of results. Concerning the inputs classifiers of our approach, we tried to use individual HD BCs trained merely on two completely independent circumstances in order to simulate the general domain knowledge and allow better variability within the individual experts. However, one can comment on the diversity of the classifiers to be minimal, especially in the case of CTG-based prediction, where the combined Bcs are learned from the same environment. This comment is actually, in favor of our approach since this latter is based on the diversity principle. Despite the lack of a large diversity of individual classifiers, the proposed approach succeeded achieving a high performance. The obtained results support that, with larger diversity our approach will be able to achieve higher performance. A second concern is related to the context-data size. We assume that the context data has to be representative rather than large, a property that has to be investigated. In the four performed experiments, the context datasets were chosen randomly and their sizes ranged from 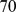 to 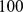 in the case of HD prediction, and equal to 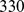 in the case of CTG-based prediction. These sizes are relatively small ones, but we can not say that they are not representative. Such a claim needs more analysis of the data density with respect to size, as well as to other data features in both HD and CTG problems. Although in the CTG context size is relatively reasonable, we believe that our approach has to be experimented with larger context data assuming that, the more the data the better the context representation.

**Figure 2:**
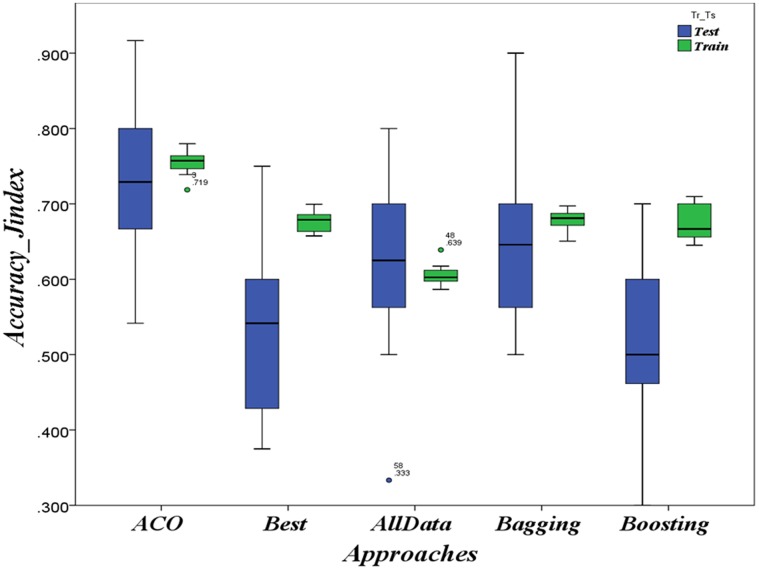
Evaluation in HD case: Prediction accuracies in the context of *Cleveland* population 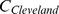 ACO-based approach Vs. Best model, data-combination Model, Boosting and Bagging.

**Figure 3:**
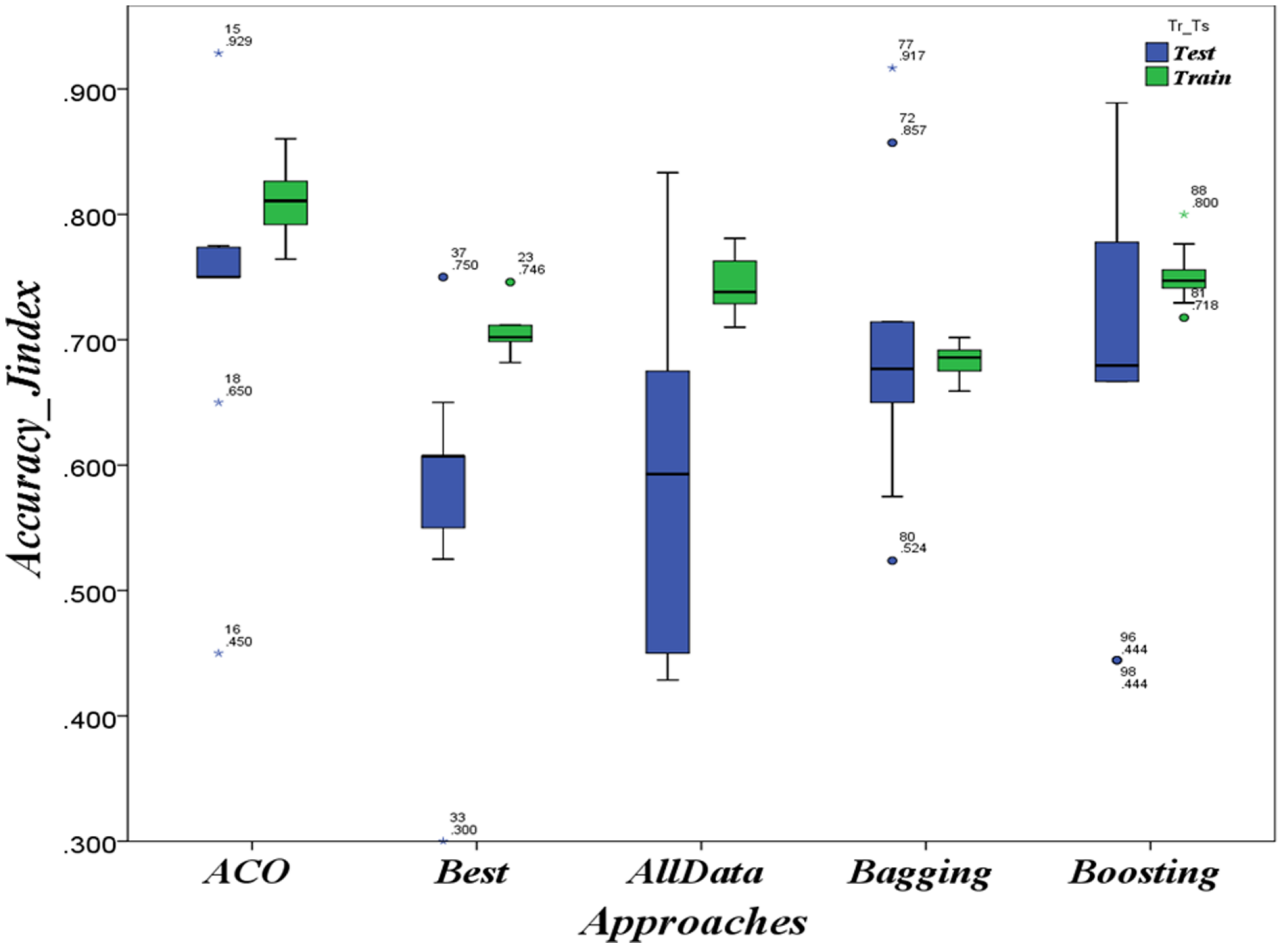
Evaluation in HD case: Prediction accuracies in the context of *Hungarian* population 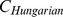 ACO-based approach Vs. Best model, data-combination Model, Boosting and Bagging.

**Figure 4:**
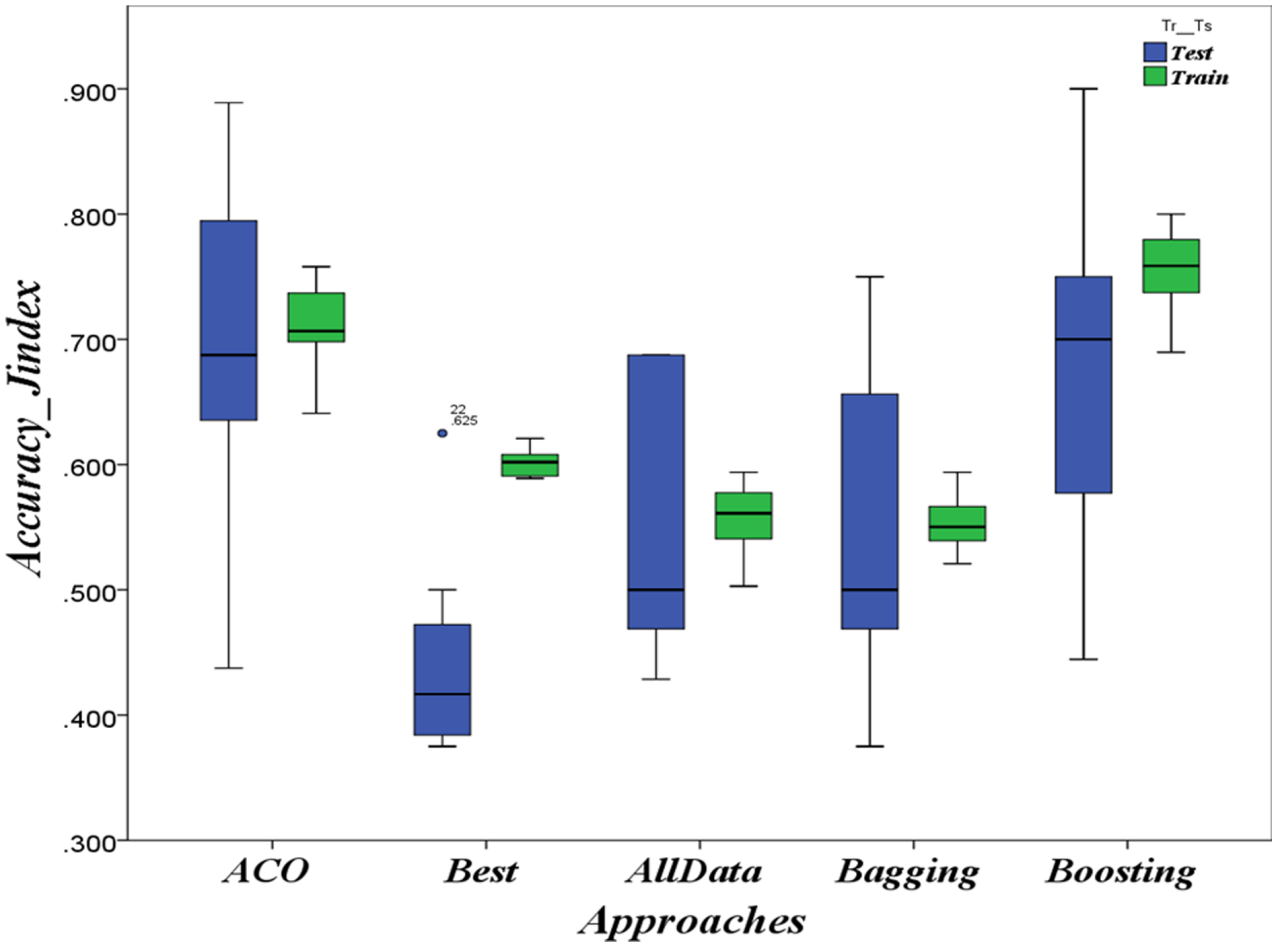
Evaluation in HD case: Prediction accuracies in the context of *Long-Beach* population 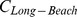 ACO-based approach Vs. Best model, data-combination Model, Boosting and Bagging.

**Figure 5:**
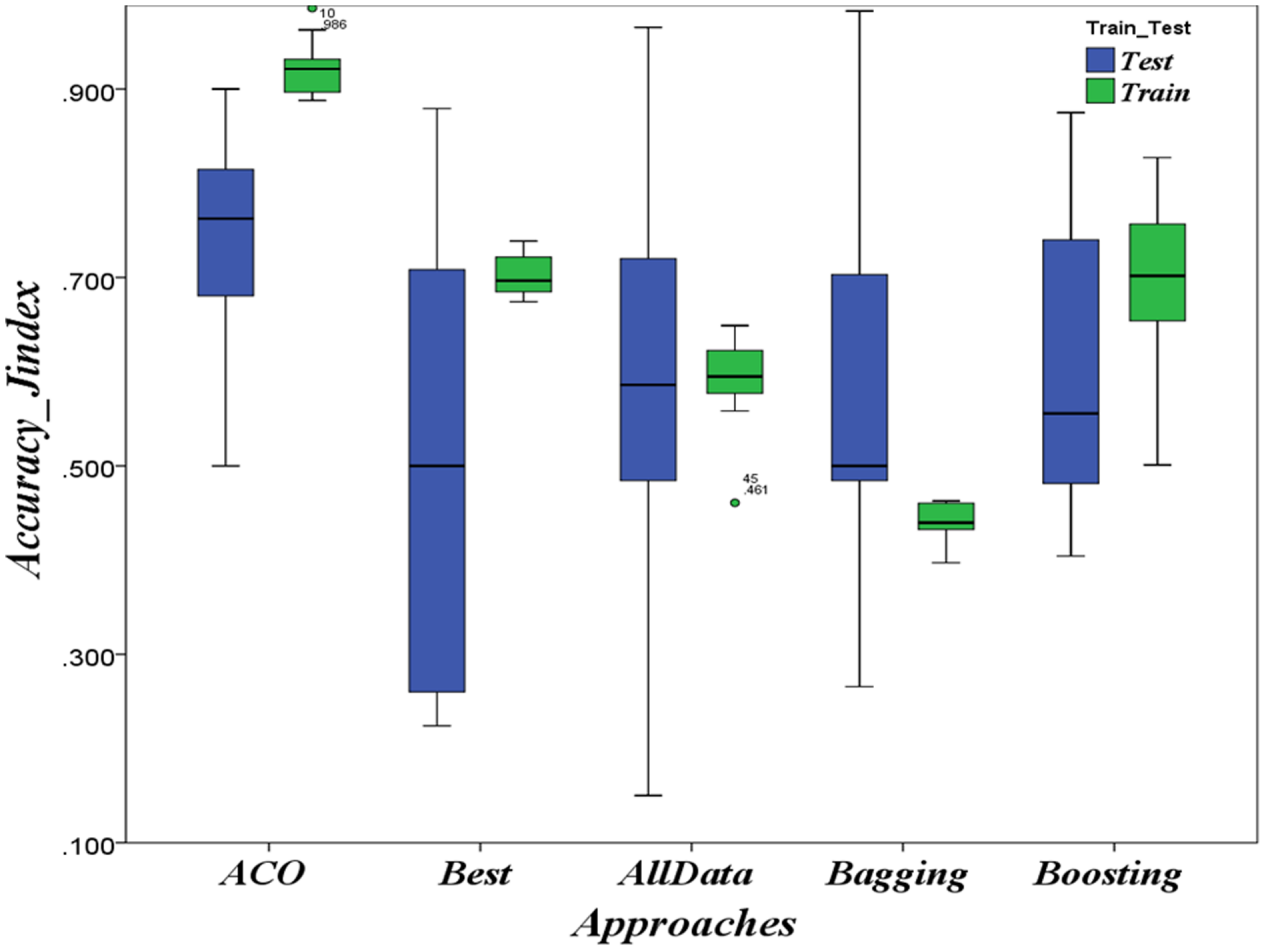
Evaluation in CTG case: Prediction accuracies in the CTG context, ACO-based approach Vs. Best model, data-combination Model, Boosting and Bagging.

The results of applying ACO-based approach on the three HD contexts Hungarian, Cleveland and Long-Beach as well as on the CTG context are respectively summarized in Figures 2, 3, 4 and 5 as boxplots charts. The accuracies boxplots on both training and testing data are grouped in the chart by the benchmark approaches in the following order: 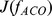, 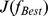, 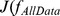), 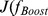) and 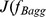).

The proposed approach follows the trends of predictions in many domains. In particular, these trends aim at promoting interpretability which is gaining an increasing interest. We share the belief that the prediction model or classifier should have the ability to explain its predictions and exhibit the causality relationships between the inputs and the outputs. Without an attached semantic or a potential of explaining, the prediction is hard to be accepted. In the field of healthcare management, Physicians need to calculate and analyze various factors in order to diagnose and prevent accurately the threats to human health. Certainly, they need to understand the causality mechanism with which they identify the risk factors responsible for undesirable health problems such as heart disease and fetal pathologies. The interpretability of the resulting classifiers allows a such mandatory understanding. Indeed, by simply looking at the attribute compositions of the final resulting BCs, we can easily interpret the link between the classifier’s inputs and its outputs. Therefore, we can draw the following interpretations:

1. Some attributes are always keeping almost the same conditional probability distribution over many final resulting BCs obtained by several runs. In other words, these attributes are built-up of a set of stables expertise chunks learned by our approach. These stable expertise chunks can resist to the context evolution and give a better generalization ability to the prediction model.
2. The attributes where the conditional probability distribution is near-uniform, have to be carefully studied and even considered as bad predictors; A first example of such an attribute in the problem of CTG prediction is the *FM*’s attribute (i.e., the number of fetal movements per second) and a second example in the problem of HD prediction is the *CHOL*’s attribute (i.e., serum cholesterol in mg/dl). Both attributes keep a near-uniform distribution of conditional probabilities in all the derived classifiers.
3. The attributes that are build-up of stables expertise chunks and with conditional probability distribution constantly different from a normal one can be considered as good predictors.
4. By exploring the resulting BCs of many runs of our algorithm for both HD and CTG problems, we realized that the *FHRBL*’s attribute (i.e., Baseline fetal heart rate) keeps the most stable conditional probability distribution; it is mostly the same non-uniform distribution over all the derived classifiers. Hence, we classify the attribute *FHRBL* as a good CTG-based predictor of the fetal health. Similarly, by interpreting the HD classifiers we discovered that *CPT*’s attribute (i.e., Chest Pain Type) is a good predictor of HD in a patient.

These interpretations suggest that some attributes could not be good predictors of the targeted health problem in both HD and CTG-based predictions. Although, these results require more validation by experts and clinicians, the above conclusions show that our approach can, in part, substitute a feature selection technique.

Our approach has demonstrated an outperformance over all the alternative approaches including ECM based methods. Our experiment is subject to threats to validity. According to the validity classification of Cook and Campbell [44], we to discuss the internal, external and construct threats to the validity of results. The primary issue that affects the internal validity of our controlled experiments is instrumentation. In our case, several programs and tools were required to conduct the experiment, including the machine learning tools, the data collection programs and the ACO tool of BC combination. These tools can add variability and negatively affect our experiment. To reduce this threat, we chose a high quality tool to build Bayesian classifiers and implemented a reliable ACO algorithm further tested with inputs of different scales. A second issue affecting internal validity is the model accuracy evaluation choice and whether it yields what it claims to measure. As discussed in Section 4.2.5 Youden’s *J-index* is well suited to classification problems in health care domain, where data is likely to be unbalanced with respect to the health problem to predict. The accuracy function was well-defined and also tested on a wide set of classifiers.

Threats to external validity limit the ability to generalize the results of the experiment to industrial practice. In order to avoid such threats, we applied our approach to two different prediction problems namely, HD disease and Cardiotography-based predictions. Four experiments were conducted in four different contexts. In each experiment, the proposed ACO algorithm was applied on a completely different and unseen dataset collected in different locations in the world. In addition, the performance of our approach achieved in each context is compared with state of the art ECM methods. Nevertheless, it is necessary to replicate the application of our approach on problems from different fields whenever data is available. Besides, applying our approach to other types of models will strengthen its generalizability. To avoid problems that affect our ability to draw correct conclusions, we used tests with high statistical power and rigorous techniques to estimate results; in particular, we precisely estimated classifier accuracy using 10-fold cross-validation. Null hypotheses were rejected, in all the independent studied contexts, with strong significance levels in the medical field, i.e., an error rate lower than 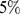 with the *t*-test.

## Conclusion

We proposed a particular solution based on ACO for a new idea of combining prediction models. Unlike the traditional ways of model combination, our idea does not consist in combining the models’ outputs but it rather combines structural elements within the models. In fact, the new idea and subsequently the particular solution are based on collecting the best chunks of expertise buried in individual existing models and combining them with respect to given circumstances. The combination process is driven by data reflecting the context where the resulting prediction model will be applied. The combinatorial complexity of our solution was helped by an ACO algorithm customized for combining Bayesian Classifiers. We applied the proposed solution to two prediction problems, namely, the heart disease and the cardiotography-based predictions. The evaluation of the ACO-based approach in four different contexts has shown promising results. In particular, the Bayesian Classifier derived by our approach performs significantly better than both the best existing expert and the expert built on all the “available data”. For the sake of valid contribution, our approach is compared to two well-known ensemble classifiers methods namely Boosting and Bagging. The results clearly show, in all the contexts, that the proposed ACO-based approach is at least as good as the Boosting and Bagging methods. With respect to the second objective of this work, i.e. the interpretability, the resulting classifiers of our approach show a potential of explaining their predictions. In particular, by enabling the selection of good predictors. Finally, the transparency of the learned clinical knowledge can help in deciding upon the appropriate treatment for heart disease or fetal pathologies, and improving the communication with patients. Future work will be devoted to the application of our approach on larger context data on the one hand, and on larger diversity of individual classifiers learned on data collected from different populations on the other hand. Furthermore, this new approach raises many new research question about its application to other types of model and to other prediction problems. Finally, a better calibration of the used ACO algorithm is needed to derive higher resulting model performance.
